# Eukaryotic RNA Polymerases: The Many Ways to Transcribe a Gene

**DOI:** 10.3389/fmolb.2021.663209

**Published:** 2021-04-21

**Authors:** Marina Barba-Aliaga, Paula Alepuz, José E. Pérez-Ortín

**Affiliations:** ^1^Instituto de Biotecnología y Biomedicina (Biotecmed), Universitat de València, València, Spain; ^2^Departamento de Bioquímica y Biología Molecular, Facultad de Ciencias Biológicas, Universitat de València, València, Spain

**Keywords:** RNA pol I, RNA pol II, transcription, nucleus, evolution, RNA pol III

## Abstract

In eukaryotic cells, three nuclear RNA polymerases (RNA pols) carry out the transcription from DNA to RNA, and they all seem to have evolved from a single enzyme present in the common ancestor with archaea. The multiplicity of eukaryotic RNA pols allows each one to remain specialized in the synthesis of a subset of transcripts, which are different in the function, length, cell abundance, diversity, and promoter organization of the corresponding genes. We hypothesize that this specialization of RNA pols has conditioned the evolution of the regulatory mechanisms used to transcribe each gene subset to cope with environmental changes. We herein present the example of the homeostatic regulation of transcript levels *versus* changes in cell volume. We propose that the diversity and instability of messenger RNAs, transcribed by RNA polymerase II, have conditioned the appearance of regulatory mechanisms based on different gene promoter strength and mRNA stability. However, for the regulation of ribosomal RNA levels, which are very stable and transcribed mainly by RNA polymerase I from only one promoter, different mechanisms act based on gene copy variation, and a much simpler regulation of the synthesis rate.

## Introduction

A key step in the central dogma of molecular biology is the transcription of pieces of DNA information into RNA molecules, which will, in some cases, be translated into proteins but will remain, in other cases, as functional non-coding RNAs (ncRNAs). In all living systems, the transcription of cellular genomes is carried out by cellular multisubunit DNA-dependent RNA polymerases (RNA pols). Eubacteria and archaea possess a single such enzyme, while eukaryotes carry out nuclear transcription with at least three RNA pols with functional specialization by each one transcribing different non-overlapping subsets of genes. Although all these enzymes have originated from a common ancestral enzyme, the increasing complexity of genomes, cells, and organisms has imposed the evolution of transcription machineries to more sophisticated systems in terms of composition, interactions, selection of target genes, and regulation. In this mini review, we summarize the presumed evolutionary origin and functional reasons that have led to the multiplicity of nuclear RNA pols in eukaryotes, and its consequences for their regulation and the homeostasis of their different RNA products. We finally focus on the different adaptation of transcription regulation by eukaryotic RNA pols to changes in cellular volume. Other eukaryotic RNA pols aspects have been extensively reviewed elsewhere (Hahn, 2004; Dieci et al., 2007; Cramer et al., 2008; Werner and Grohmann, 2011; Engel et al., 2013; Cramer, 2019).

## The Evolutionary Scheme of RNA Pol From Eubacteria to Eukaryotes

**FIGURE 1:**
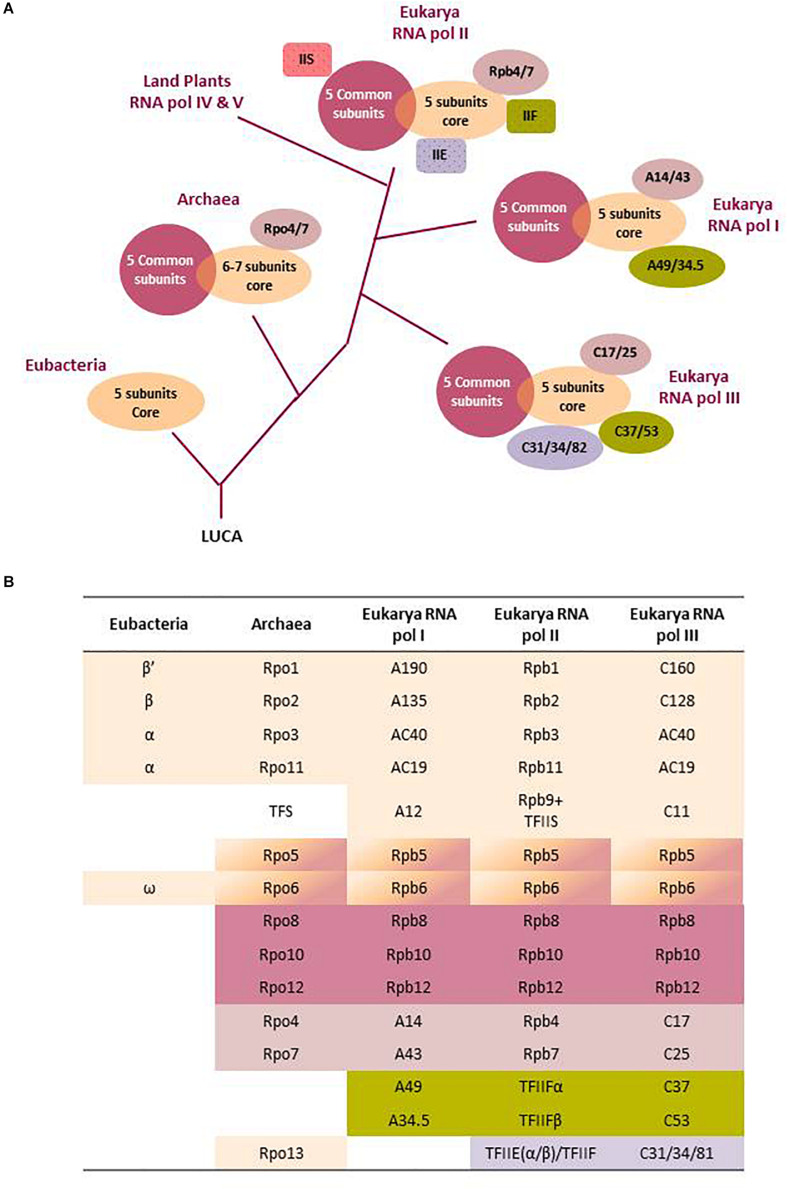
Evolutionary history and subunit organization of nuclear eukaryotic RNA polymerases. **(A)** The last universal common ancestor (LUCA) of all organisms is assumed to have a multisubunit DNA-dependent RNA polymerase. Nowadays, all living beings have RNA pols with a core of five to seven subunits. After *Eubacteria* separation, the common ancestor of *Archaea* and *Eukarya* added additional peripheral subunits. Finally, after eukaryote emergence, the *Archaea*-derived nucleus started to develop specialized RNA polymerases. Specialized RNA pols I and III integrated some transcription factors as permanent subunits which, in RNA pol II, remain independent (TFIIS, TFIIF, TFIIE). RNA pol IV and V are not fully described. Only the branching after RNA pol I separation is indicated. See the main text for further descriptions. **(B)** The table shows a comparative scheme of the RNA pol subunits aligned according to sequence and/or functional homology. Colors correspond to the structural scheme of part **(A)**. Note that the Rpb5 and 6 subunits are part of both the core and the five unit sets of common subunits to all three eukaryotic RNA pols. Archaeal Rpo13 has no equivalent in eukaryotes, and the TFS from *Archaea* is an independent homologous factor to eukaryotic TFIIS. See Werner and Grohmann (2011); Vannini and Cramer (2012), and Huang et al. (2015) for more details on RNA pol subunit structure and evolution.

All RNA pols, from eubacteria to higher eukaryotes, share basic mechanistic functioning: use of a DNA template, processive translocation on the template during RNA synthesis, utilization of ribonucleoside triphosphate as substrates, Watson–Crick base pairing of the new added nucleotide with the complementary one in the template DNA, and formation of a new phosphodiester bound by a metal-dependent mechanism. To perform these basic functions, all RNA pols contain two largest subunits (Figure 1) with double-ψβ-barrel motifs that create an active site at the interface of the subunits with three key aspartic residues conserved across all domains of life. Additionally, multisubunit RNA pols contain a variable number of additional smaller subunits (Figure 1). The two largest catalytic subunits of RNA pols are thought to have evolved from the duplication and diversification of a gene that encoded a protein cofactor of a common ancestral ribozyme, which performed RNA polymerase activity in the primal RNA world (Iyer et al., 2003). At some point of evolution, the new protein heterodimer would have gained polymerase activity and acquired different subunits with specialized assembly and auxiliary functions. Thus, all multisubunit RNA pols share a common structural core and similar basic molecular mechanisms and must derive from the RNA pol of the last universal common ancestor (LUCA) of archaea, eubacteria, and eukaryotes, assumed to have existed 3.5–3.8 billion years ago (Burton, 2014). This ancestral multisubunit RNA pol was probably similar to the simple RNA pol found today in eubacteria, which is formed (see Figure 1) by two large β and β’ catalytic subunits, two assembly subunits (2α), and one auxiliary subunit (ω), as all these five subunits are highly conserved in the structure/function of all organisms (Werner, 2007; Werner and Grohmann, 2011).

RNA pol gained greater complexity in terms of acquiring new subunits following the split of the eubacterial and archaeal–eukaryotic branches from the universal tree of life (Werner, 2007; Spang et al., 2015). Archaeal RNA pol has three or four catalytic polypeptides and three assembly and auxiliary subunits, which are closely related to bacterial ones (Figure 1). However, archaeal RNA pol has gained five additional periphery subunits with no homologs in eubacteria but resembling eukaryotic subunits, which stabilize the interactions of polymerase with template DNA, newly synthesized RNA, and different transcription factors to ensure efficient functioning in the transcription cycle (Werner, 2007; Werner and Grohmann, 2011; Fouqueau et al., 2017). The more complex transcription machineries of archaea and eukaryotes are linked with the fact that their genomes, which differ from the eubacterial genome, are stabilized and compacted by histone or histone-like proteins that impose more restrictive access to DNA and the need for additional basal transcription factors (Reeve, 2003; Geiduschek and Ouhammouch, 2005; Kwapisz et al., 2008; Jun et al., 2011; Werner and Grohmann, 2011; Koster et al., 2015).

Archaeal and eukaryotic lineages diverged more than 2 billion years ago, with eukaryotes originating from an archaeal linage with already diverse eukaryotic signature proteins (Spang et al., 2015). Other important differences include that eukaryotes have an extended system of intracellular membranes that compartmentalizes the intracellular space, and the cellular volume is three to four orders of magnitude larger than that of archaea and bacteria (Lane and Martin, 2010; Koonin, 2015). They also contain organelles (mitochondria and chloroplasts) that derive from two kinds of eubacteria and have their own RNA pol (De Duve, 2007). The most prominent difference for nuclear transcription that arises with eukaryotes is diversification into three different nuclear RNA pols with specialized functions: RNA pol I is responsible for the synthesis of a single transcript, namely, precursor ribosomal RNA, which is processed into 28S, 5.8S, and 18S rRNAs; RNA pol II synthesizes a wide diversity of transcripts, including protein-coding messenger RNA (mRNA) and many ncRNAs, such as microRNAs (mi), small nuclear (sn), and small nucleolar (sno) RNAs; RNA pol III synthesizes diverse transfer RNA (tRNA) and 5S rRNA, and also U6 small nuclear RNA and other non-coding small RNAs (Dieci et al., 2007). There are two additional nuclear RNA pols in plants (IV and V), involved in the transcription of ncRNAs that are required for transcriptional gene silencing via the RNA-directed DNA methylation (Zhou and Law, 2015). In this review, we will focus on the structure and function of RNA pols I, II, and III.

The most well-studied eukaryotic RNA pols are those of the budding yeast *Saccharomyces cerevisiae*, and it is thought that they are good models for other eukaryotic RNA pols. For this reason, we use the names of yeast RNA pols genes and subunits throughout this review (Figure 1). Yeast RNA pols I, II, and III have a structurally conserved horseshoe-shaped core formed by 10 subunits (Figure 1) homologous to archaeal RNA pol subunits and a different number of additional periphery eukaryote specific subunits (Darst, 2001; Werner, 2007; Cramer et al., 2008). The 10 subunit cores include the two largest catalytic subunits (the two upper rows in Figure 1B), five additional subunits (Rpb5, 6, 8, 10, and 12) common to the three nuclear RNA pol, the A12/Rpb9/C11 subunit involved in proofreading (see below) and the AC40–AC19 heterodimer, shared between RNA pols I and III and homologous to Rpb3–Rpb11 in RNA pol II (Fernández-Tornero et al., 2013). The additional periphery yeast RNA pol subunits are mostly essential for cell viability but are not strictly required for RNA polymerization. Instead, they increase the regulatory potential and allow the specialization of each RNA pol in the transcription of a non-redundant subset of genes (Werner, 2007; Cramer et al., 2008; Koster et al., 2015). RNA pol II has a dissociable dimer (Rpb4/7) that plays important roles during the multifaceted transcription elongation of this RNA pol. This dimer has a homology with the Rpo4/7 dimer of archaeal RNA pol and has a counterpart (with low homology) in the A14/A43 and C17/C25 dimers of RNA pols I and III, respectively (Figure 1). RNA pol I has a further dimer (A49/A34) that has an equivalent in RNA pol III (C37/C53) but is not a constitutive part of RNA pol II where its function is conducted by the independent TFIIF factor (α/β dimer; Vannini and Cramer, 2012). This dimer plays a specific role in the particular mode of initiation of all three RNA pols (Abascal-Palacios et al., 2018) and in RNA pol III termination (Hoffmann et al., 2015; Arimbasseri and Maraia, 2016) that very much differs from the other two RNA pols in this stage (Proshkina et al., 2006; Werner and Grohmann, 2011). RNA pol III has an additional and totally specific trimer (C31/C34/C82) that is homologous to RNA pol II TFIIE and is proposed to be involved in the mechanism of RNA pol III initiation (Hoffmann et al., 2015). This trimer has been proposed to be TFIIF–TFIIE hybrid rather than simply a TFIIE-like subcomplex (Abascal-Palacios et al., 2018).

The coexistence of the conserved, but different, largest core subunits of the three RNA pols (A190/A135, Rpb1/Rpb2, and C160/C128 in RNA pols I, II, and III, respectively) in all eukaryotes is remarkable and suggests their early evolutionary divergence. At the same time, the substantial conservation of the central RNA pol core since LUCA indicates that it performs essential processes required for gene expression that allows very little innovation. Therefore, in order to generate complex eukaryotes, most evolutionary innovation is expected to occur in periphery subunits, especially in RNA pol II, which specifies the cellular proteome that confers unique characteristics to different cell types through mRNA synthesis. Additionally, the unique *C*-terminal domain (CTD) of the largest catalytic subunit (Rpb1) of RNA pol II is also one source for innovation in mRNA transcription regulation and a mark of the eukaryotic lineage (Burton, 2014). CTD consists of a repeating structure that is rich in serine and other phosphorylable amino acids, which increases in number of repetitions with greater evolutionary complexity. Another consequence of eukaryotic innovation is the complex structure of RNA pol III with 17 subunits, which are all conserved to a certain degree in eukaryotes from yeast to humans. This supports the notion of the early divergence of RNA pol III from RNA pols I and II (Proshkina et al., 2006; Figure 1). Of all these considerations, it can be suggested that the last eukaryote common ancestor is likely to have already had distinct RNA pols I, II, and III, as well as the repetitive structure at the CTD of RNA pol II (Proshkina et al., 2006; Yang and Stiller, 2014). It can be concluded that the existence and evolution of the three specialized RNA pols in eukaryotic cells would have allowed the division of labor and enabled intricate gene regulation in multicellular complex organisms that requires the cell cycle, tissue-specific, environmental, and developmental regulation of gene expression (Dieci et al., 2007; Cramer, 2019). RNA pols IV and V are thought to have evolved more recently from RNA pol II through subfunctionalization of silencing activities performed by RNA pol II in fungi and metazoans in the earliest land plants (Huang et al., 2015).

## Differences in the Three RNA Pol Structure Linked to Differences in Function

Although the transcription cycle (initiation, elongation, and termination) has similar principles in all three nuclear RNA pols, the specific features of their transcription modes are reflected in their subunit structures. RNA pol II targets a large set of differently regulated genes, which requires the capacity to interact with a bigger set of transcription initiation and elongation factors than the other two RNA pols. Perhaps this was accomplished by having less permanent subunits than the other two RNA pols, but by also having dissociable subunits (Rpb4/7) and independent initiation and elongation factors (TFIIF, TFIIS, and TFIIE), while the equivalent factors in other polymerases form an intrinsic part (subunits) of the RNA pol complex. For example, RNA pol I has a single promoter to recognize but requires high-speed, efficient elongation to avoid collisions between polymerases in its highly crowded genes (Goodfellow and Zomerdijk, 2013). This is perhaps the reason why RNA pol I possesses important intrinsic RNA cleavage activity for proofreading and a rapid resumption of elongation after pausing (Fernández-Tornero et al., 2013). This activity resides in its A12 subunit with homology to both the RNA pol II Rpb9 subunit and the TFIIS elongation factor. Thus, A12 seems to be a fusion protein that comprises the amino-terminal domain of the RNA pol II Rbp9 subunit and the carboxy-terminal domain of TFIIS (Hoffmann et al., 2015). A similar reasoning can be done for RNA pol III where the C11 subunit has homology to Rpb9 and TFIIS (Chédin et al., 1998). The more complicated process of resuming elongation after pausing in RNA pol II suggests the need for specific regulation, which is not required for simpler and faster RNA pol I/III elongation (Engel et al., 2013). Another example of functions that fall in RNA pol III intrinsic subunits but in external transcription factors in RNA pol II is related to transcription termination. RNA pol III specific dimer (C53/C37) together with C11 subunit are particularly required for the very fast efficient termination and coupled re-initiation needed by this RNA pol due to the highly transcribed and very short genes that it targets (Dieci et al., 2013; Arimbasseri and Maraia, 2016). In fact, RNA pol III termination is distinct from that of the other two nuclear RNA pols because its genes present a tract of oligo-T at the 3′ end, which induces termination. On the contrary, RNA pols I and II require additional *cis*-acting elements and ancillary factors for termination (Arimbasseri and Maraia, 2016). In short, both RNA pols I and III seem to have integrated some transcription factor-like subunits into the core enzyme during evolution to prioritize rapid efficient transcription *versus* regulation (Carter and Drouin, 2010).

Chromatin imposes a major limitation to transcription by three eukaryotic RNA pols preventing their direct targeting to gene promoters, which probably explains why all nuclear RNA pols are first engaged in pre-initiation complexes before starting transcription. Pre-initiation complexes minimally consist of the TATA box-binding protein (TBP), which is common to all three transcription systems, initiation factors TFIIB (RNA pol II) and Brf1 (RNA pol III), and the RNA pol II-specific TFIIE factor (Hahn, 2004; Naidu et al., 2011). Moreover, during elongation, chromatin imposes clearly different conditions to each RNA pol. Active rRNA genes are totally covered by transcribing RNA pol I complexes to form characteristic Christmas trees with no nucleosomes (Albert et al., 2012; Goodfellow and Zomerdijk, 2013). Most RNA pol III genes (tRNAs and 5S, especially) are so short that the whole transcribing unit lies in a short track free of nucleosomes (Shukla and Bhargava, 2018), unlike RNA pol II that transcribes longer genes and deals with nucleosomes during elongation. The arrest and backtracking of RNA pol II occur at nucleosome barriers, and elongation is resumed by the stimulation of weak intrinsic RNA pol II cleavage activity by TFIIS to form a new RNA 3′ end in its active site (Cramer, 2019). This more complicated way of solving backtracking could serve to refine the elongation regulation process (Bradsher et al., 1993; Shilatifard et al., 1996).

## The Need for Coordination of the Three RNA Pol Activities: the Case of Translation Machinery

Translation machinery (ribosomes and tRNAs) synthesis requires the tight coordination among all nuclear RNA pols because rRNAs are synthesized by RNA pols I and III and ribosomal proteins are made from mRNAs transcribed by RNA pol II. Hence their coordination at all times and in all growth regimes is clearly necessary. The existence of five common subunits and one universal initiation factor, TBP, in all three RNA pols may be used to establish common regulatory mechanisms for nuclear transcription. RP mRNAs are some of the most abundant mRNAs in actively growing cells (Pelechano et al., 2010), and, thus, their synthesis forms a significant part of the total RNA pol II effort (Warner, 1999). Moreover, many other RNA pol II genes encode proteins involved in ribosome biogenesis but are not part of ribosomes. These include the RNA pol I and III subunits and the proteins involved in rRNA and tRNA maturation, and transport and translation factors, which are coordinately regulated (RiBi regulon in yeast) and also share some regulatory mechanisms with RP genes (Martin et al., 2006; Bosio et al., 2017). Therefore, the coordination of ribosome biogenesis and its regulation by growth must require the existence of regulatory mechanisms that coordinate their output. Candidates for this role are mammalian c-Myc and the yeast Sfp1 transcription factors (Lempiäinen and Shore, 2009). Regulation of RNA pols by growth is dependent on the target of rapamycin and Ras–PKA pathways that link ribosome production to nutrient availability (Warner, 1999; Martin et al., 2006; Mayer and Grummt, 2006; Lempiäinen and Shore, 2009). These pathways act by regulating the activity of several transcription activators, such as Rap1, Abf1, or Sfp1, in yeast (see Bosio et al., 2017, for further details).

## An Example of Different Regulation of RNA Pols Related to Their Different Function: Ribostatic Control During Cell Volume Variations

The different properties of eukaryotic RNA pols and their RNA products predict that the regulatory mechanisms used by each one to cope with changes will be different. We discuss here an example that we have recently studied in yeast *S. cerevisiae*: the regulation of global RNA pol I and II activities with respect to changes in cell volume.

Homeostasis is defined as the state of steady internal conditions maintained by living beings and includes the control of concentrations of cell molecules. The terms ribostasis and proteostasis refer to the modulation of RNA and protein levels, respectively, in response to changes in the environment. Proteins are mostly the final goal of gene expression and are in charge of catalytic and structural functions. For this reason, their homeostasis is very strictly controlled, and the total protein concentration remains quite constant (Liebermeister et al., 2014; Milo and Phillips, 2015; Benet et al., 2017). Nonetheless, gene expression regulation occurs chiefly at the mRNA level. For this purpose, mRNAs are mostly unstable, and the overall mRNA concentration is controlled within a certain range (Pérez-Ortín et al., 2013; Benet et al., 2017). On the contrary, rRNAs and tRNAs remain very stable during active growth and only degrade under stress conditions or when defects in the molecule occur (Deutscher, 2006; Pérez-Ortín et al., 2019).

Homeostasis deals with the molecular concentration, and not with the number of molecules. Therefore, changes in the cell volume are expected to provoke adaptation mechanisms to maintain homeostasis. In yeast cells, and probably in other organisms, volume varies depending on the genotype, the cell cycle phase (Jorgensen et al., 2002; Ferrezuelo et al., 2010), aging (Egilmez et al., 1990), ploidy (Cook and Tyers, 2007; Lee et al., 2009), and the growth rate (Aldea et al., 2017). To maintain ribostasis and proteostasis, increases in cell volume must be compensated by the coordinated increase in the amounts of RNA molecules and proteins (Bustamante et al., 2014; Walters and Parker, 2015).

Studies carried out in different model organisms have established differences between transcription regulatory responses to cell volume depending on the organism and the RNA pol studied and suggest the existence of a size-sensing mechanism that produces alterations in transcription (Wu et al., 2010). Changes in the RNA pol II transcription rate (TR) with volume increase have been widely studied to show that it is differentially regulated in cells with different cell division types. Thus, for symmetrically dividing cells, such as mammalian fibroblasts (Padovan-Merhar et al., 2015), or *Schizosaccharomyces pombe* (Zhurinsky et al., 2010), RNA pol II nascent TR increases in parallel with volume due to a bigger and faster recruitment of polymerase onto chromatin (Sun et al., 2020). Thus, for symmetrically dividieng cells, such as *S. cerevisiae*, nascent TR remains constant by controlling the expression of RNA pol II coding genes, while mRNA stability increases to maintain mRNA ribostasis (Mena et al., 2017). This difference is explained by asymmetric cell division in *S. cerevisiae* resulting in two cells with different volumes: a small daughter cell and a large mother cell. In this scenario, the strategy adopted by eukaryotes with symmetric cell division, such as *S. pombe* or fibroblasts, is not applicable, as it would result in a higher mRNA net synthesis rate in small daughter cells (Mena et al., 2017). However, the strategy adopted to adapt ribostasis to increased cell volume is very different for RNA pol I. In this case, nascent TR increases with cell volume by increasing the number of copies of the rDNA gene. The higher gene copy number can occur by increasing cell ploidy or by expanding the number of rDNA repeats (Mena et al., 2017; Pérez-Ortín et al., 2021). This mode of regulation is a slow form of TR regulation because changes in the genome can occur only during replication (Kobayashi, 2006, 2011; Nelson et al., 2019).

Why is there a different solution for an identical problem in RNA pols I and II? We hypothesize that the differences in their targets, the 35S gene, and protein-encoding genes conditioned the evolution of different regulatory mechanisms for RNA pol I and RNA pol II. As the rRNA TR needs to reach much higher levels than that of any of the RNA pol II genes, eukaryotic cells evolved a specialized faster polymerase with a single gene template with many repeated copies. RNA pol is able to form extremely dense head-to-tail “camel caravans” in which the A49 subunit from one molecule contacts directly with A43 from the neighboring molecule. Thus, the specialized dimer A49/A43 allows a higher RNA pol loading rate than in RNA pol II (Albert et al., 2011). On the other hand, the repeated nature of the rDNA locus is prone to cause homologous recombination (Iida and Kobayashi, 2019) and offers the opportunity to alter the rDNA copy number and, thus, total TR without changing nascent TR per gene copy. In this way, RNA pol I can be controlled in the short term at the transcription initiation and elongation levels, as with other RNA pols, but also in the long term by changing its copy number during genome replication (Kobayashi et al., 1998; Pérez-Ortín et al., 2021). An interesting question arises here: what happens to the RNA pol III that transcribes tRNAs and 5S genes, whose gene number is also a few hundred copies (Turowski and Tollervey, 2016)? Interestingly, 5S genes are localized within the rDNA repeats in the genome of *Saccharomycotina* clade (Bergeron and Drouin, 2008), which comprises mostly asymmetrically dividing yeasts, which could imply a common TR regulation strategy for RNA pols I and III in rRNA synthesis. To support this idea, in other yeasts and most of other eukaryotes with symmetric cell division, 5S genes are usually dispersed along the genome (Drouin and De Sa, 1995).

To summarize, eukaryotes differentiate from prokaryotes not only because of a more complex intracellular organization with nuclear and organelle evolutionarily independent genomes but also because the unprecedented job division occurs between several distinct nuclear RNA pols. The specialization of each one in the synthesis of a specific subset of transcripts with different abundance, stability, and function has forced differences in transcription initiation, elongation, termination, and regulation strategies but has provided, at the same time, the versatility to make phenotypically different cells from the same genome as a requisite for multicellular organisms.

## Author Contributions

JP-O conceived the manuscript. PA, MB-A, and JP-O wrote the manuscript. All authors contributed to the article and approved the submitted version.

## Conflict of Interest

The authors declare that the research was conducted in the absence of any commercial or financial relationships that could be construed as a potential conflict of interest.
